# Serum metabolomics of hyperbilirubinemia and hyperuricemia in the Tibetan plateau has unique characteristics

**DOI:** 10.1038/s41598-023-40027-6

**Published:** 2023-08-07

**Authors:** Heng Zhang, Xianzong Ma, Junfeng Xu, Peng Jin, Lang Yang, Yuanming Pan, Fumei Yin, Jie Zhang, Jiheng Wang, Dongliang Yu, Xiaoying Wang, Mingjie Zhang, Xin Wang, Dezhi Wang, Jianqiu Sheng

**Affiliations:** 1 Medical School of Chinese PLA, Beijing, 100853 China; 2 Department of Gastroenterology, The Seventh Medical Center of Chinese PLA General Hospital, No.5 Nanmencang, Beijing, 100700 China; 3 Senior Department of Gastroenterology, The First Medical Center of Chinese PLA General Hospital, Beijing, 100853 China

**Keywords:** Biochemistry, Environmental sciences, Diseases, Health occupations, Pathogenesis

## Abstract

Few studies have provided data on the metabolomics characteristics of metabolic diseases such as hyperuricemia and hyperbilirubinemia in the Tibetan plateau. In the current study, we sought to investigate the serum metabolomics characteristics of hyperbilirubinemia and hyperuricemia in the Tibetan plateau, with the aim to provide a basis for further research on their pathogenesis, prevention, and treatment. The study participants were born in low-altitude areas below 1000 m and had no prior experience living in a high-altitude area before entering Golmud, Tibet (average elevation: 3000 m) and Yushu, Qinghai (average elevation: 4200 m). Thirty-four participants with hyperbilirubinemia (18 in Golmud and 16 in Yushu), 24 participants with hyperuricemia, and 22 healthy controls were enrolled. The serum samples of subjects were separated and then sent to a local tertiary hospital for biochemical examination. Serum widely targeted technology, based on the ultra-performance liquid chromatography tandem mass spectrometry (UPLC-MS/MS) platform, was used to detect serum metabolites and differential metabolites. Compared to the healthy controls, hyperbilirubinemia patients from Golmud showed 19 differential metabolites, hyperbilirubinemia patients from Yushu showed 12 differential metabolites, and hyperuricemia patients from Yushu showed 23 differential metabolites. Compared to the hyperbilirubinemia patients from Golmud that is at a low altitude, the Yushu groups had 33 different metabolites. Differential metabolites are primarily classified into amino acids and their derivatives, nucleotides and their derivatives, organic acids and their derivatives, and lipids/fatty acids. These are related to metabolic pathways such as caffeine metabolism, arachidonic acid metabolism, and tyrosine metabolism. Hyperbilirubinemia and hyperuricemia in the Tibetan plateau have unique serum metabolomics characteristics. Glycine derivatives and arachidonic acid and its derivatives were associated with plateau hyperbilirubinemia, and vanillic acid and pentadecafluorooctanoic acid were associated with plateau hyperuricemia.

## Introduction

In medicine, a plateau environment refers to an area that is 3000 m or more above sea level, which has the characteristics of low oxygen partial pressure, cold climate, high wind speed, and strong ultraviolet rays^1^. Moreover, when people from the plains rapidly enter a plateau, acute mountain sickness (AMS) is likely to occur due to hypoxia. A series of nonspecific clinical syndromes, such as headache, dizziness, nausea, vomiting, insomnia, and fatigue, may occur in mild cases, while in severe cases, AMS will cause damage to the heart, lung, brain, and other important organs^2–5^. Furthermore, high altitude exposure could decrease splanchnic perfusion^6^ and blood oxygen levels, leading to hypoxia and hypoxia-induced reductive oxidative stress^7^.

Bilirubin is the primary metabolite of iron porphyrin compounds; hyperbilirubinemia refers to a situation in which the total bilirubin level is more than 20.5 mol/L, which may occur due to excessive red blood cell death, the reduced ability of hepatocytes to convert bilirubin, or blocked bilirubin excretion^8^ and can cause irreversible damage to the nervous system^9^. Bilirubin is an important antioxidant that can scavenge reactive oxygen species (ROS) and reduce the level of oxidative stress in the body^10^. Previous studies have found that the level of heme oxygenase-1 (HO-1) in the blood of climbers was significantly increased, which can catalyze the production of biliverdin, iron, and CO from heme^11^. Subsequently, biliverdin is reduced to bilirubin, resulting in increased bilirubin levels in the body. Hyperuricemia refers to a fasting blood uric acid level greater than 420 μmol/L in men and 360 μmol/L in women under a normal purine diet. Previous studies have found that the level of uric acid (the end product of purine metabolism in the body) was significantly increased in people in the plateau^12,13^. As the level of uric acid increases, the ROS-RAS pathway can be activated, resulting in pro-oxidative stress^14,15^. Hypoxia can cause liver injury through elevated oxidative stress and cell apoptosis at high altitudes^16,17^. The liver is the direct bilirubin-producing organ and a major uric acid-producing site^10,18^. Liver injury caused by hypoxia at high altitudes can result in direct bilirubin entering the bloodstream. Xanthine oxidase, which is mainly present in the liver and spleen, is a key rate-limiting enzyme for uric acid production^18^. Hypoxia-induced liver injury could enhance the expression of xanthine oxidase, increasing uric acid^19^.

In recent years, metabolomics has developed rapidly to study the pathogenesis of metabolic diseases. Among them, widely targeted metabolomics integrates the advantages of non-targeted and targeted metabolite detection technologies. Metabolomics can achieve high-throughput, high-sensitivity, wide-coverage, and accurate metabolite detection and analysis by using high-sensitivity liquid chromatography–tandem mass spectrometry (LC–MS/MS) and a self-built metabolite database. It possesses unique advantages for revealing the metabolic processes and the pathogenesis of diseases. Therefore, in this study, we aim to explore the metabolomic characteristics and pathogenesis of hyperbilirubinemia and hyperuricemia at high altitudes using widely targeted metabolomics combined with clinical laboratory indicators.

## Participants and methods

### Study participants

A total of 614 soldiers who grew up in plain areas and had entered high-altitude areas in Golmud (377 persons, average altitude: 3000 m) and Yushu (237 persons, average altitude: 4200 m) for the first time were selected as a group to conduct an epidemiological questionnaire survey and blood biochemical examination. Following the collection of blood samples from the soldiers, the samples were left undisturbed for 30 min, before being centrifuged at 3000 rpm at 4 °C for 15 min. Subsequently, serum samples were separated and sent to a local tertiary hospital for biochemical examination. The inclusion criteria were hyperbilirubinemia (total bilirubin > 20.5 μmol/L, 18 cases in Golmud, 16 cases in Yushu) and hyperuricemia (> 420 μmol/L in males or > 360 μmol/L in females, 24 cases), while the other biochemical indicators were normal. Twenty-two healthy participants residing in the plateau were used as health controls.

### Ethics statements

This study was approved by the Ethics Committee of the Seventh Medical Center of Chinese PLA General Hospital (protocol number: 2018069). Informed consent was collected from all participants. All experiments were conducted in accordance with Good Clinical Practice (GCP) and with the ethical principles of the Declaration of Helsinki.

### Sample preparation and extraction

The samples stored at − 80 °C were thawed on ice and vortexed for 10 s. Subsequently, 50 μL of samples and 300 μL of extraction solutions (ACN: methanol = 1:4, V/V) containing internal standards were added into a 2-mL microcentrifuge tube. The samples were vortexed for 3 min and then centrifuged at 12,000 rpm for 10 min (4 °C). Next, 200 μL of the supernatant was collected and placed at − 20 °C for 30 min, before being centrifuged at 12,000 rpm for 3 min (4 °C). Finally, 180 μL aliquots of supernatant were transferred for LC–MS analysis.

### Conditions of high-performance liquid chromatography (HPLC)

The sample extracts were analyzed using an LC–ESI–MS/MS system (UPLC, ExionLC AD, https://sciex.com.cn/; MS, QTRAP®6500+, https://sciex.com/). The analytical conditions were as follows: UPLC: column, Waters ACQUITY UPLC HSS T3 C18 (1.8 µm, 2.1 mm × 100 mm); column temperature, 40 °C; flow rate, 0.4 mL/min; injection volume, 2 μL; solvent system, water (0.1% formic acid): acetonitrile (0.1% formic acid); gradient program, 95:5 V/V at 0 min, 10:90 V/V at 11.0 min, 10:90 V/V at 12.0 min, 95:5 V/V at 12.1 min, 95:5 V/V at 14.0 min.

### Electrospray ionization quadrupole-linear ion trap mass spectrometry/mass spectrometry (ESI-QTRAP-MS/MS)

Linear ion trap (LIT) and triple quadrupole (QQQ) scans were acquired on a triple quadrupole-linear ion trap mass spectrometer (QTRAP), QTRAP® LC–MS/MS System, equipped with an ESI Turbo Ion-Spray interface, operating in positive and negative ion modes and controlled by Analyst 1.6.3 software (Sciex). The ESI source operation parameters were as follows: source temperature, 500 °C; ion spray voltage (IS), 5500 V (positive), − 4500 V (negative); ion source gas I (GSI), gas II (GSII), and curtain gas (CUR) were set at 55, 60, and 25.0 psi, respectively; the collision gas (CAD) was high. Declustering potential (DP) and collision energy (CE) for individual multiple reaction monitoring mode (MRM) transitions were measured with further optimization. A specific set of MRM transitions was monitored for each period according to the metabolites eluted within this period.

### Statistical analysis

The statistical analysis was performed using SPSS 26.0 (IBM, USA). The measurement data are expressed as $\overline{{\text{x}}}$ ± s, and the comparison between groups was performed using *t-test* or nonparametric test. The enumeration data are expressed as the rate (%), and the comparison between groups was performed using the χ^2^ test. A P-value < 0.05 was considered statistically significant. A qualitative analysis of blood metabolites from 80 participants on the plateau was conducted based on the UPLC-MS/MS detection platform, Metware Database (MWDB), and the metabolite information public database. The MRM of triple four-pole mass spectrometry was used for quantitative analysis of metabolites, while multiple regression analysis methods, such as principal component analysis (PCA), partial least squares discriminant analysis (PLS-DA), and orthogonal partial least squares discriminant analysis (OPLS-DA), were used to identify and analyze the changes in each metabolite. Based on the OPLS-DA results, the metabolites with fold changes ≥ 2 and ≤ 0.5 were selected. If there were biological repeats in the sample grouping, the metabolites with variable importance in projection (VIP) ≥ 1 were selected based on the above. The results were combined with the Kyoto Encyclopedia of Genes and Genomes (KEGG) database^20,21^ and the human metabolome database (HMDB) to search for differential metabolite-related metabolic pathways and disease information.

### Ethics approval and consent to participate

This study was approved by the Ethics Committee of the Seventh Medical Center of Chinese PLA General Hospital (Protocol Number 2018069). Informed consent was collected from all participants. All experiments were conducted in accordance with Good Clinical Practice (GCP) and the ethical principles of the Declaration of Helsinki.

## Results

### Analysis of the incidence of gastrointestinal symptoms in the Tibetan plateau participants and the biochemical characteristics of the research participants

As shown in Table 1, the participants were all males aged 19–28 years, with a local residence time of > 2 years. The average age of the participants in the healthy control group was 22.90 ± 0.66 years, the average age of the participants in the hyperbilirubinemia group was 24.71 ± 0.86 years, and the average age of the participants in the hyperuricemia group was 21.29 ± 0.60 years. The levels of both direct and indirect bilirubin were upregulated in the hyperbilirubinemia group compared to healthy controls at high altitudes. The hyperuricemia group exhibited significantly enhanced levels of uric acid and direct bilirubin, whereas the indirect bilirubin production was unchanged. Additionally, the levels of alanine aminotransferase (ALT) were elevated in both the hyperbilirubinemia and hyperuricemia groups compared to the controls.

**Table 1:** Clinical and demographic characteristics of the study participants.

Index	Healthy control (n = 22)	HB (n = 34)	P-value	HU (n = 24)	P-value
Age (years) (mean ± SD)	22.90 ± 0.66	24.71 ± 0.86	0.14	21.29 ± 0.60	0.08
Residence time (years) (mean ± SD)	3.40 ± 0.52	4.53 ± 0.66	0.22	3.11 ± 0.43	0.66
BMI (kg/m^2^) (mean ± SD)	21.56 ± 1.39	22.01 ± 0.45	0.73	20.75 ± 0.36	0.59
Total bilirubin (μmol/L) (mean ± SD)	13.34 ± 0.88	28.09 ± 1.21	0.00	18.57 ± 1.45	0.00
Direct bilirubin (μmol/L) (mean ± SD)	4.64 ± 0.27	10.55 ± 0.95	0.00	9.65 ± 1.10	0.00
Indirect bilirubin (μmol/L) (mean ± SD)	8.70 ± 0.68	17.54 ± 1.30	0.00	8.81 ± 0.86	0.92
Serum uric acid (μmol/L) (mean ± SD)	365.50 ± 6.87	390.82 ± 13.10	0.15	488.75 ± 8.80	0.00
Alanine aminotransferase (U/L) (mean ± SD)	23.91 ± 1.67	36.18 ± 3.86	0.02	49.04 ± 7.81	0.00
Aspartate aminotransferase (U/L) (mean ± SD)	21.09 ± 0.93	26.99 ± 2.93	0.12	37.04 ± 8.90	0.10

*HB* hyperbilirubinemia, *HU* hyperuricemia.

### Analysis of the metabolic signatures of patients with plateau hyperbilirubinemia

**Figure 1:**
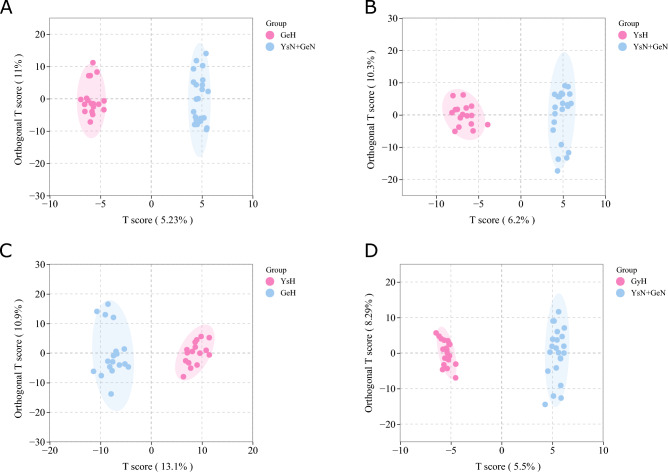
Orthogonal partial least squares discriminant analysis (OPLS-DA) score plot map. (**A**) Golmud hyperbilirubinemia (GeH) vs. healthy control. (**B**) Yushu hyperbilirubinemia (YsH) vs. healthy control. (**C**) GeH vs. YsH. (**D**) Hyperuricemia group vs. healthy control.

**Figure 2:**
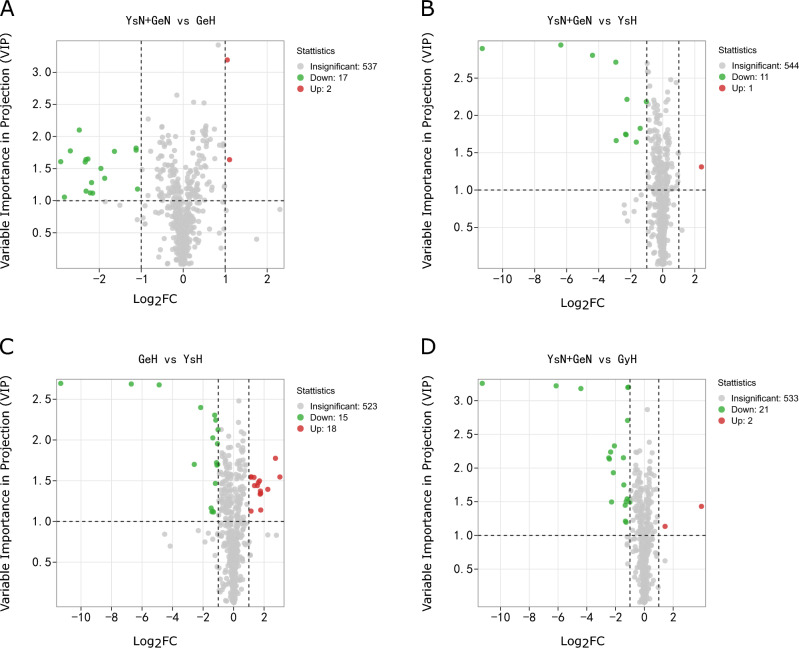
Volcano plot of differential metabolites among groups. (**A**) Golmud hyperbilirubinemia (GeH) vs. healthy control. (**B**) Yushu hyperbilirubinemia (YsH) vs. healthy control. (**C**) GeH vs. YsH. (**D**) Hyperuricemia group vs. healthy control. Each point in the volcano plot represents a metabolite, the abscissa represents the logarithm of the quantitative difference between two samples of a metabolite, and the ordinate represents the VIP value. The green dots in the figure represent downregulated differentially expressed metabolites, the red dots represent upregulated differentially expressed metabolites, and the black dots represent metabolites that were detected but not significantly different.

**Figure 3:**
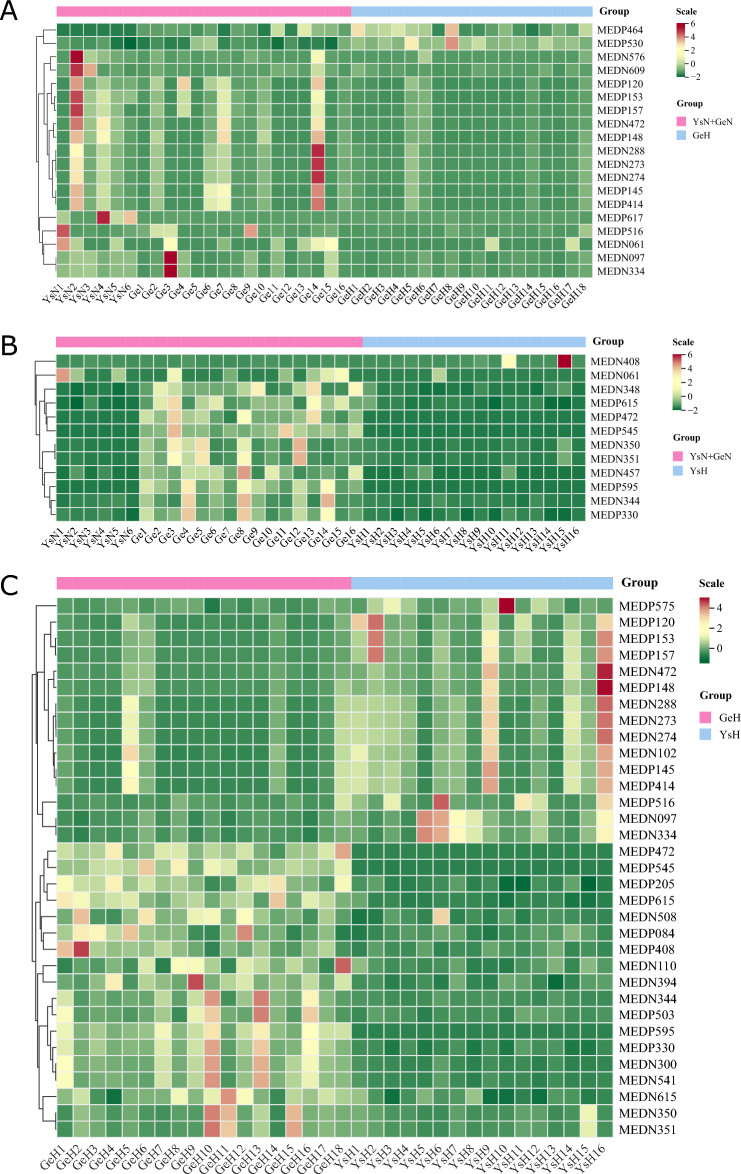

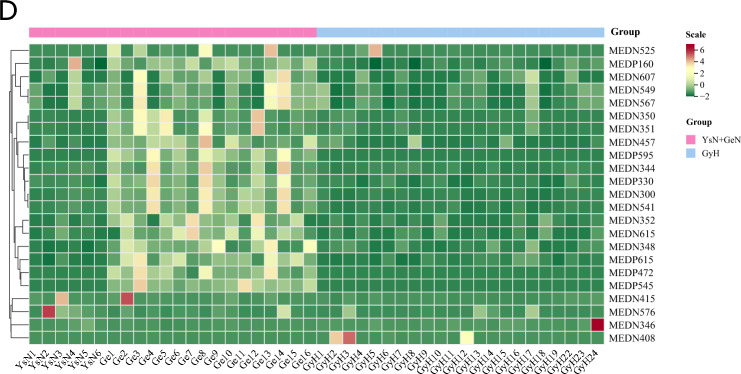
Clustering heatmap of differential metabolites. (**A**) Golmud hyperbilirubinemia (GeH) vs. healthy control. (**B**) Yushu hyperbilirubinemia (YsH) vs. healthy control. (**C**) GeH vs. YsH. (**D**) Hyperuricemia group vs. healthy control.

**Figure 4:**
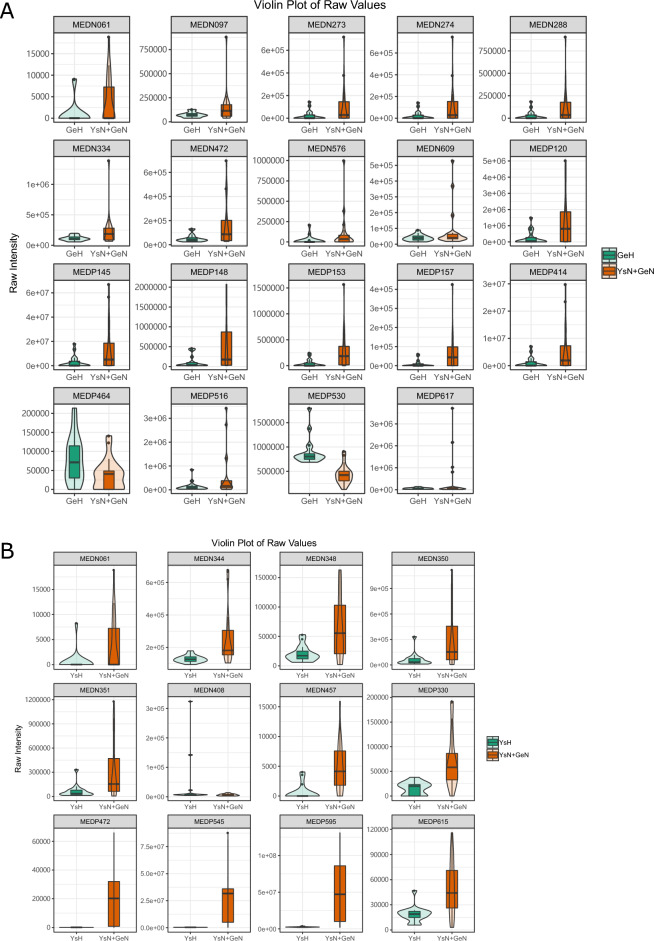

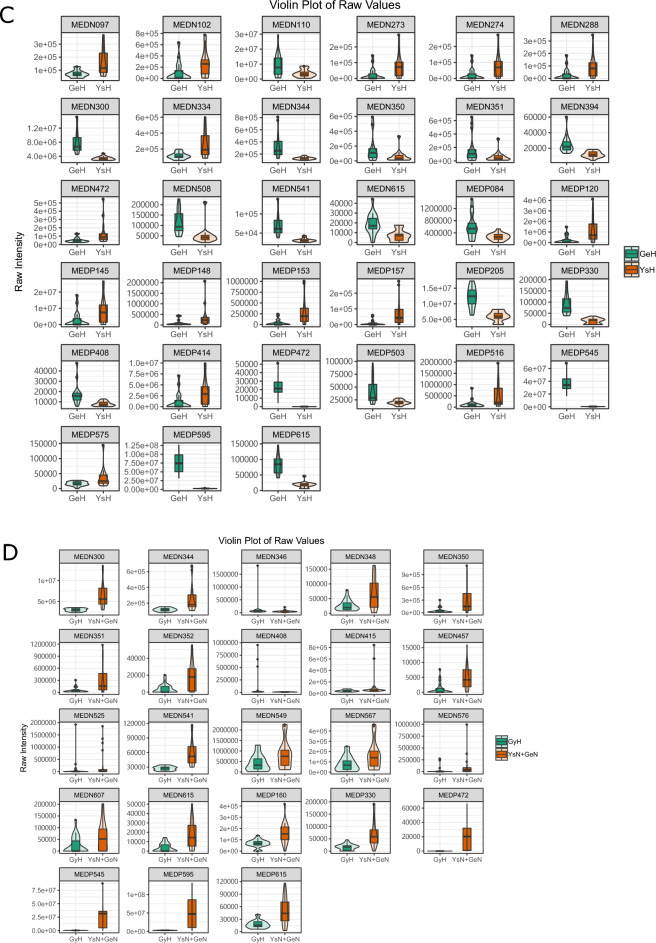
Violin plot of differential metabolites. (**A**) Golmud hyperbilirubinemia (GeH) vs. healthy control. (**B**) Yushu hyperbilirubinemia (YsH) vs. healthy control. (**C**) GeH vs. YsH. (**D**) Hyperuricemia group vs. healthy control.

The results of OPLS-DA are shown in Fig. 1. Compared to the healthy control group, the Golmud and Yushu groups had obvious tendencies to separate metabolites (Fig. 1A,B). Compared to the Golmud group, the Yushu group had an obvious tendency to separate metabolites (Fig. 1C). A total of 556 metabolites were detected. Compared to healthy controls, 17 metabolites were downregulated and two were upregulated in the Golmud group, as indicated by the volcanic map analysis (Fig. 2A). Additionally, 11 metabolites were downregulated and one metabolite was upregulated in the Yushu group (Fig. 2B). Compared to the Golmud group, the Yushu group had 18 downregulated and 15 upregulated metabolites (Fig. 2C). The differential metabolic clustering heatmap is shown in Fig. 3A–C, and the metabolites among the groups showed obvious clustering. The violin plot of the differential metabolites showed that the content of 15-deoxy-δ-12,14-PGJ2, hydroxyphenethylamine significantly increased (VIP > 1), while the content of arachidonic acid (AA), 1,3-dimethyluric acid, 1,7-dimethyluric acid, 3,7-dimethyluric acid, mandelic acid, 1-methyluric acid, aminophylline, uridine triphosphate (UTP), 1,7-dimethylxanthine, 1-methylxanthine, 3-methylxanthine, 7-methylxanthine, theobromine, P-hydroxyphenyl acetic acid, 1,2,3-trihydroxybenzene, *N*-phenylacetylglycine, and 2-furoylglycine decreased significantly (VIP > 1) in the Golmud group compared to the healthy control group (Fig. 4A). The content of pentadecafluorooctanoic acid (PFOA) in the Yushu group was significantly increased (VIP > 1), while thromboxane B2, 15-hydroxyeicosatetraenoic acid (15-hete), 12-hydroxyeicosatetraenoic acid (12-hete), suberic acid, sebacate, 1-*O*-feruloylquinic acid, 2-pyrrolidone, 5-methyl-THF, oxymetazoline, *N*-phenylacetylglycine, and oxidized glutathione were significantly downregulated (VIP > 1) (Fig. 4B). Organic acids and their derivatives, nucleotides and their derivatives, pyridine and its derivatives, and benzoic acid and its derivatives were significantly increased in the Yushu group compared to the Golmud group (Fig. 4C).

### Analysis of the metabolic signatures of patients with plateau hyperuricemia

The results of OPLS-DA are shown in Fig. 1D. The metabolite separation trend in the hyperuricemia group was obvious compared to that in the healthy control group. A total of 556 metabolites were detected. Compared to the healthy controls, two metabolites were upregulated and 21 were downregulated in the hyperuricemia group, as shown by the volcano plot analysis (Fig. 2D). The differential metabolic clustering heatmap is shown in Fig. 3D, and the metabolites in the hyperuricemia group and the healthy controls showed obvious clustering. The violin plot of the differential metabolites (Fig. 4D) showed that compared to the healthy control group, the hyperuricemia group had significantly higher contents of PFOA and vanillic acid (VA) (VIP > 1), while o-phosphoethanolamine, thromboxane B2, 15-hete, 12-hete, azelaic acid, subericacid, sebacate, carbamoyl phosphate, 1-*O*-feruloyl quinic acid, 5-methyl-THF, 2-pyrrolidone, adenosine, 2-(formylamino) benzoic acid, p-cresol, o-cresol, DL-3,4-dihydroxyphenyl glycol, chloramphenicol, salicylic acid, 1,2,3-trihydroxybenzene, oxymetazoline, and oxidized glutathione were significantly decreased (VIP > 1). The primary differential metabolites were amino acids and their derivatives, nucleotides and their derivatives, organic acids and their derivatives, and lipids/fatty acids. Among them, PFOA increased, and 1-*O*-feruloylquinic acid and 2-pyrrolidone decreased significantly. Additionally, the changes in the organic acids and their derivatives were relatively obvious among the three groups, and 1-*O*-feruloylquinic acid and 2-pyrrolidone decreased most significantly between the Yushu group and healthy control group and between the Golmud group and Yushu group.

### Pathway analysis of differential metabolite enrichment

**Figure 5:**
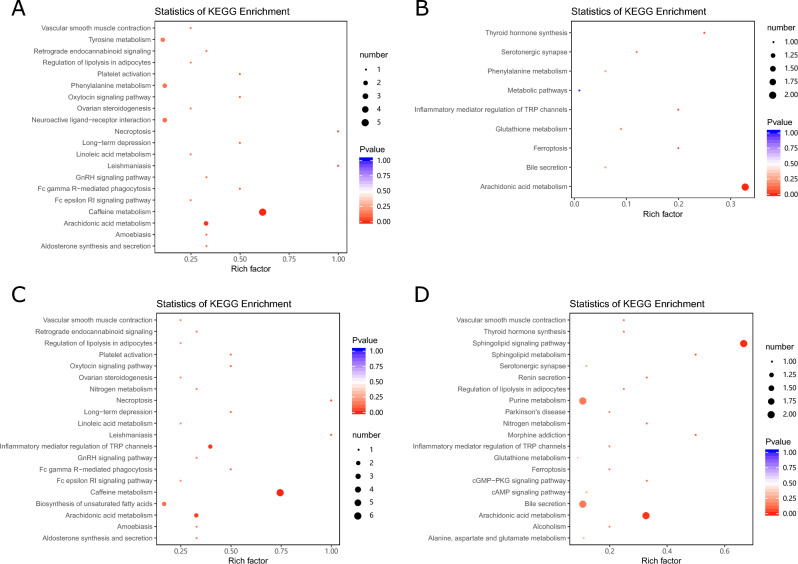
KEGG enrichment map of differential metabolites. (**A**) Golmud hyperbilirubinemia (GeH) vs. healthy control. (**B**) Yushu hyperbilirubinemia (YsH) vs. healthy control. (**C**) GeH vs. YsH. (**D**) Hyperuricemia group vs. healthy control. The abscissa represents the rich factor corresponding to each pathway, the ordinate is the pathway name, and the color of the point is the P-value. The size of the dots represents the number of enriched differential metabolites.

Enrichment analysis of the metabolic pathways involved in the differential metabolites showed that compared to the healthy controls, the metabolic pathways enriched by the serum differential metabolites in the Golmud group were primarily the metabolic pathways, caffeine metabolism, tyrosine metabolism, phenylalanine metabolism, AA metabolism, and the neuroactive ligand–receptor interaction. Compared to the healthy controls, AA metabolism and bile secretion were the primary metabolic pathways enriched in the serum differential metabolites in the Yushu group. Compared to the Golmud group, the primary metabolic pathways of the serum differential metabolites enriched in the Yushu group were caffeine metabolism, inflammatory mediator regulation of the transient receptor potential (TRP) channels, tyrosine metabolism, biosynthesis of the puffed fatty acids, and AA, among which, the upregulated metabolites were primarily involved in caffeine metabolism. Compared to the metabolic pathways in the healthy controls, those enriched by the differential metabolites in the plateau hyperuricemia group were primarily bile secretion, purine metabolism, AA metabolism, and the sphingolipid signaling pathway (Fig. 5).

## Discussion

In this study, we found that compared to healthy controls in the Tibetan plateau, participants with hyperbilirubinemia in Golmud had significantly higher levels of nucleotides and their derivatives, lipids and their derivatives, and some organic acid metabolites. Hyperbilirubinemia participants from Yushu showed that benzene and its derivatives, some nucleotides, organic acids, and vitamins and their derivatives were significantly reduced. Additionally, serum glycine derivatives (primarily phenylacetylglycine) and AA and its metabolites 12-hete and 15-hete found in Golmud and Yushu participants with hyperbilirubinemia were significantly reduced. Previous studies have shown that glycine could reduce ROS levels to protect cells^22^. AA is a polyunsaturated fatty acid that can induce or inhibit oxidative stress^23,24^, while oxidative stress could further facilitate the release of AA^25^. Moreover, 12-hete is the primary metabolite of AA catalyzed by 12-lipoxygenase (12-LOX), which promotes the generation of ROS and is involved in the body’s oxidative stress response^26^. Furthermore, 15-hete is a metabolite of AA catalyzed by 15-lipoxygenase (15-LOX), which can promote angiogenesis by stimulating the production of vascular endothelial growth factor (VEGF)^27–29^, improve the viability of pulmonary artery smooth muscle cells^30^, and inhibit the apoptosis of pulmonary artery smooth muscle cells through heat shock protein 90 (HSP90)^31^. Additionally, a study on neonates with hyperbilirubinemia found that they were in a state of oxidative stress and that the activity of the serum antioxidant enzymes decreased with an increase in the serum bilirubin levels^32^. Moreover, the inadequate oxygen-carrying capacity of erythrocytes could promote hemoglobin production to ensure oxygen supply, increasing indirect bilirubin in the blood. Liver injury induced by hypoxia at high altitudes could facilitate direct bilirubin entry into the bloodstream. Our study revealed that the hyperbilirubinemia group exhibited increased levels of direct bilirubin, indirect bilirubin, and ALT when compared to the control group, suggesting that the red blood cells from subjects of the hyperbilirubinemia group might fail to carry adequate oxygen and that these subjects might be more vulnerable to liver injury induced by hypoxia. Bilirubin exhibits antioxidant properties in vivo and in vitro^33,34^, and it is speculated that the ROS levels in patients with hyperbilirubinemia decreased due to the antioxidant role of bilirubin, causing a decrease in glycine derivatives and AA and its metabolites 12-hete and 15-hete^35^.

Xanthine oxidase, which is primarily present in the liver and spleen, is a key rate-limiting enzyme for uric acid production^18^. Hypoxic liver injury has been shown to elevate the expression of xanthine oxidase, increasing uric acid^19^. We observed that the hyperuricemia group exhibited elevated levels of direct bilirubin and ALT compared to the healthy controls. Therefore, we speculated that subjects in the hyperuricemia group were more susceptible to hypoxic liver injury. This study showed that compared to healthy participants residing on the plateau, the serum levels of benzene and its derivatives, lipids, and some organic acids and its derivatives in patients with hyperuricemia were significantly lower, whereas the VA and PFOA levels were significantly increased. Previous studies have shown that VA has strong antioxidant, antihypotension, and antiapoptotic functions and has protective effects on the heart and liver^36–38^. Indeed, it has been shown that cells pretreated with VA can reduce ROS production and attenuate mitochondrial-mediated caspase-3 activity, thereby reducing apoptosis in H9c2 cells after hypoxia-reoxygenation (H/R) injury^39^. The increase in direct bilirubin could reduce ROS levels in patients with hyperuricemia at high altitudes. However, elevated levels of uric acid could exacerbate ROS production and may eventually upregulate ROS levels in the body, resulting in a compensatory increase in VA levels. Moreover, we found that 1-*O*-feruloylquinic acid and 2-pyrrolidone were significantly downregulated in the serum of participants with plateau hyperbilirubinemia and hyperuricemia, although the reasons and significance still require clarification.

The results of KEGG analysis showed that compared to the healthy control group, the serum differential metabolites of patients with hyperuricemia were primarily involved in metabolic pathways, caffeine metabolism, and AA metabolism. Meanwhile, the serum differential metabolites of patients with hyperuricemia were primarily involved in metabolic pathways, bile secretion, purine metabolism, AA metabolism, and the sphingolipid signaling pathway. The pathway enrichment analysis showed that the significantly altered pathways of the serum differential metabolites in patients with hyperbilirubinemia included necroptosis, leishmaniasis, caffeine metabolism, AA metabolism, and thyroid hormone synthesis. The significantly altered pathways of serum differential metabolites in patients with hyperuricemia included the sphingolipid signaling pathway, sphingolipid metabolism, and morphine addiction. Bile secretion can affect bilirubin levels in the body, and similarly, purine metabolism can affect uric acid levels in the body. Consistent with previous conclusions, AA metabolism was associated with hyperbilirubinemia and hyperuricemia. Additionally, it is speculated that the participants in the plateau had a habit of drinking tea, so the metabolites of which are involved in the caffeine metabolism pathway.

This study was a cross-sectional investigation with some shortcomings. First, lifestyle information, such as diet and physical activity, can affect metabolism, and we did not assess these factors. However, the selected soldiers all adopted a unified recipe and ate regularly and this weakened the influence of diet on the research results to a certain extent. Second, the people selected for this study were all young soldiers who lived in the Tibetan plateau for more than 2 years and were compared to the plateau population; this may weaken the representativeness of the study results, although it better reflects the metabolomics signatures of metabolic diseases in participants residing on the plateau and provides a certain basis for their prevention and treatment research. Due to the limited availability of previous similar studies and a lack of references for sample size calculation, we maximized the inclusion of eligible subjects based on the screening criteria, without conducting sample size estimation.

## Conclusions

In conclusion, our analysis of serum metabolites in different groups revealed that glycine derivatives, as well as AA and its derivatives, are distinctive metabolites associated with hyperbilirubinemia in people living in high-altitude areas, while VA and PFOA are specific metabolites associated with hyperuricemia in people living in high-altitude areas. This study provides new perspectives and evidence for understanding the pathogenesis and prevention of hyperbilirubinemia and hyperuricemia in people living in high-altitude areas by using widely targeted metabolomics combined with clinical laboratory indicators.

## Data Availability

All materials are commercially available, and the datasets used and/or analyzed during the current study are available from the corresponding author on reasonable request.

## Abbreviations

UPLC-MS: Ultra-performance liquid chromatography tandem mass spectrometry
AMS: Acute mountain sickness
NF-κb: Nuclear factor kappa-B
HO-1: Heme oxygenase-1
ROS: Reactive oxygen species
RAS: Renin-angiotensin system
LC–MS/MS: Liquid chromatography–tandem mass spectrometry
HPLC: High performance liquid chromatography
LC: Liquid chromatography
ESI: Electrospray ionization
QTRAP: Quadrupole-linear ion trap
LIT: Linear ion trap
QQQ: Triple quadrupole
IS: Ion spray
MRM: Multiple reaction monitoring mode
DP: Declustering potential
CE: Collision energy
MWDB: Metware Database
PCA: Principal component analysis
PLS-DA: Partial least squares discriminant analysis
OPLS-DA: Orthogonal partial least squares discriminant analysis
VIP: Variable importance in projection
KEGG: Kyoto Encyclopedia of Genes and Genomes
HMDB: Human metabolome database
ALT: Alanine aminotransferase
AA: Arachidonic acid
UTP: Uridine triphosphate
PFOA: Pentadecafluorooctanoic acid
15-hete: 15-Hydroxyeicosatetraenoic acid
12-hete: 12-Hydroxyeicosatetraenoic acid
VA: Vanillic acid
TRP: Transient receptor potential
12-LOX: 12-Lipoxygenase
15-LOX: 15-Lipoxygenase
VEGF: Vascular endothelial growth factor
HSP90: Heat shock protein 90
H/R: Hypoxia-reoxygenation
